# Cation Effect in the Corrosion Inhibition Properties of Coumarate Ionic Liquids and Acrylic UV-Coatings

**DOI:** 10.3390/polym12112611

**Published:** 2020-11-06

**Authors:** Esther Udabe, Anthony Sommers, Maria Forsyth, David Mecerreyes

**Affiliations:** 1 Department of POLYMAT, University of the Basque Country UPV/EHU, 20018 Donostia-San Sebastian, Spain; esther.udabe@polymat.eu; 2 Institute for Frontier Materials, Deakin University, Geelong, VIC 3220, Australia; anthony.somers@deakin.edu.au; 3 IKERBASQUE Basque Foundation for Science, 48009 Bilbao, Spain

**Keywords:** corrosion inhibitors, ionic liquids, poly(ionic liquid)s

## Abstract

Chromate free corrosion inhibitors are searched for to mitigate the economic loss caused by mid-steel corrosion. Here, we show metal-free organic inhibitors having free coumarate anions that can be used either as direct corrosion inhibitors or incorporated into a polymer coating obtained by UV-curing. Four different ionic liquid monomers and polymer coatings with hexoxycoumarate anion and different polymerizable counter cations were investigated. Potentiodynamic polarization, electrochemical impedance spectroscopy, and surface analyses have verified their corrosion inhibition performance on a mild steel AS1020 surface. In the case of the coumarate ionic liquid monomers, the most promising inhibitor is the one coupled with the ammonium cation, showing an inhibition efficiency of 99.1% in solution followed by the imidazolium, pyridinium, and anilinium. Next, the ionic liquid monomers were covalently integrated into an acrylic polymer coating by UV-photopolymerization. In this case, the barrier effect of the polymer coating is combined with the corrosion inhibitor effect of the pendant coumarate anion. Here, the best polymer coatings are those containing 20% imidazolium and pyridinium cations, presenting a greater impedance in the EIS (Electrochemical Impedance Spectroscopy) measurements and less evidence of corrosion in the scribe tests. This article shows that the cationic moiety of coumarate based ionic liquids and poly(ionic liquid)s has a significant effect on their excellent corrosion inhibition properties for a mild steel surface exposed to aqueous chloride solutions.

## 1. Introduction

Materials deterioration by corrosion is one of the biggest technological issues nowadays causing significant economic losses [1]. Corrosion can be seen in our daily life in packaging, pipelines, infrastructure, and in chemical industries [2]. In the last decades, scientists have been researching corrosion inhibitors which adsorb onto a metal surface and decrease the effects of corrosion processes by suppressing the anodic and/or cathodic electrochemical reactions. The most used inhibitor is that based on well-known hexavalent chromium compounds that creates a complex oxide layer on the steel surface to act as a barrier to corrosion [3]. However, due to the high toxicity of hexavalent chromium inhibitors, new, environmentally friendly, and yet efficient corrosion inhibitors are being sought [4,5].

Over the years many different organic and organometallic compounds have been investigated as corrosion inhibitors [6,7]. The mechanism of action of organic corrosion inhibitors is based on the adsorption onto the surface to form a protective film which displaces water from the metal and protects it against deterioration due to attack by aggressive species such as chloride ions. This process is neither physical nor purely chemical adsorption, but usually a combination of both processes [8,9,10]. Effective organic corrosion inhibitors usually contain heteroatoms (nitrogen, oxygen, sulfur or phosphorous) with lone electron pairs and moieties with π-electrons (aromatic rings and multiple bonds) that can interact with free d-orbital of the metal, favoring the adsorption process [6,7]. In addition, ionic charges as well as long aliphatic chains usually have a beneficial role in the adsorption process of the inhibitors [11,12]. One very promising family of metal-free, low toxicity organic inhibitors recently demonstrated revolves around tailored ionic liquids [11]. As an example, Chong et al. reported ionic liquids based on imidazolium cations and aromatic carboxylate anions showing an effective corrosion inhibition for mild steel. Among the different ionic compounds, 2-methylimidazolinium p-coumarate was shown to be a most effective inhibitor, showing a strong anodic inhibition effect [13,14,15,16,17]. The p-coumarate anion has also been shown to be effective when coupled to various other cations, including rare earth metals [1,9,17].

Another very efficient tool of protection against corrosion is the use of polymer coatings that isolate the metal from the corrosive environment. Nowadays, the use of a barrier coating or primer on the metal surface can complement the use of an active corrosion inhibitor. Polymer coatings based on polyurethanes, epoxy resins, polyesters, fluorinated polymers, or polyacrylates are commonly used in industrial applications [18]. The development of advanced coatings which include further functionalities, such as superhydrophobicity, conductive polymers, graphene, or hybrid coatings, are a topic of current research [18]. Interestingly, organic inhibitors can be combined onto a polymer coating as additives into the coating formulation. However, the use of additives shows limitations due to the difficult migration of the inhibitor to the metal surface or the potential leaching out of the polymer coating to the environment [19]. Thus, the chemical incorporation of the inhibitor into the polymer is an interesting strategy. One way to achieve this is to polymerize the inhibiting moiety into the coating by developing monomeric ionic liquid inhibitors. Therefore, we have initiated a program on the development of poly(ionic liquid)s corrosion inhibitors as a new application for this emerging family of functional polymers which are finding applications in emerging technologies, such as batteries, (bio)electronics, gas separation membranes, or water purification [20,21,22,23,24].

Very recently, we reported new methacrylic ammonium coumarate ionic liquids that show great potential as corrosion inhibitors [25]. Interestingly those methacrylic monomers could be incorporated into an UV-curable acrylic polymer coating showing highly effective corrosion protection of mild steel. In our previous article, we compared new methacrylic ammonium ionic liquids having three different coumarate counter-anions p-substituted having hydroxy, butoxy, and hexoxy groups [25]. Among them, the hexoxy coumarate derivative showed the highest inhibition efficiency due to the beneficial effect of the hydrophobic tail. For this reason, in this article we fixed the hexoxy coumarate anion and we compared the effect of the polymerizable counter-cation. The goal of this article is to determine the effect of the chemical structure of the cation of the monomer on the corrosion inhibition properties of hexoxy coumarate based monomeric ionic liquids. Potentiodynamic polarization, electrochemical impedance spectroscopy experiments and surface analysis were carried out to verify and compare the corrosion inhibition performance of the monomers on the mild steel AS1020 surface. The incorporation of the hexoxy coumarate based cationic monomers into acrylic polymers obtained by UV-photopolymerization was proposed as a means to deliver efficient coatings against mild steel corrosion.

## 2. Materials and Methods

### 2.1. Materials

p-Coumaric acid, potassium hydroxide and Darocur (Speedcure 73) were obtained from Sigma Aldrich, city, country. 1-Bromohexane was obtained from Acros, Geel, Belgium. Oxybis(propane-1,2-diyl) diacrylate, dipropylene glycol diacrylate, trimetylpropyl triacrylate, cyclic trimethylolpropane formal acrylate and acid-based adhesion promotors were gifted by Arkema/Sartomer, Colombes, France. Mild Steel AS1020, NaCl aqueous solution, HCl aqueous solution, MiliQ water, methanol, and ethanol were used without further purification.

### 2.2. Monomer Synthesis

#### 2.2.1. Synthesis of (E)-3-(4-(Hexyloxy)Phenyl)Acrylic Acid, 2

**Scheme 1:**
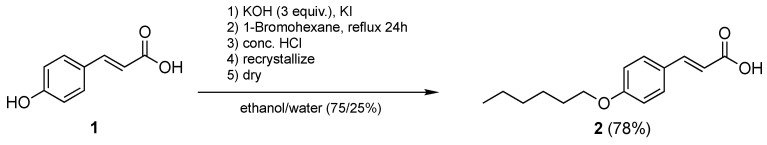
Synthesis of (E)-3-(4-(hexyloxy)phenyl)acrylic acid, 2, from p-coumaric acid, 1.

Commercially available p-coumaric acid was used as a starting material for the synthesis of (E)-3-(4-(hexyloxy)phenyl)acrylic acid 3. In presence of KOH and KI, p-coumaric acid 1 reacted with 1-bromohexane (1 equivalent) to produce a potassium salt 2. Solvent was removed and compound 2 was acidified with concentrated HCl. The crude product was recrystallized from a mixture of ethanol/water. The final product 2 was dried under vacuum and obtained as a white powder in a good yield (78%) (Scheme 1).

(E)-3-(4-(hexyloxy)phenyl)acrylic acid 3. Yield 78%; ^1^H NMR (D_2_O) δ (ppm): 0.85 (t, 3 H, CH_3_), 1.30 (m, 4 H, CH_3_(CH_2_)_2_), 1.42 (m, 2 H, O(CH_2_)_2_CH_2_), 1.74 (m, 2 H, OCH_2_CH_2_), 4.08 (t, 2 H, OCH_2_), 6.36 (d, 1 H, CH=CHCOO), 6.99 (d, 2 H, Ar–H), 7.31 (d, 2 H, Ar–H), 7.55 (d, 1 H, CH=CHCOO)

#### 2.2.2. Synthesis of 2-(Dimethyl Ammonium)Ethyl Methacrylate p-Hexoxy Coumarate 3, [DMAEM+ HexOCou-]

**Scheme 2:**
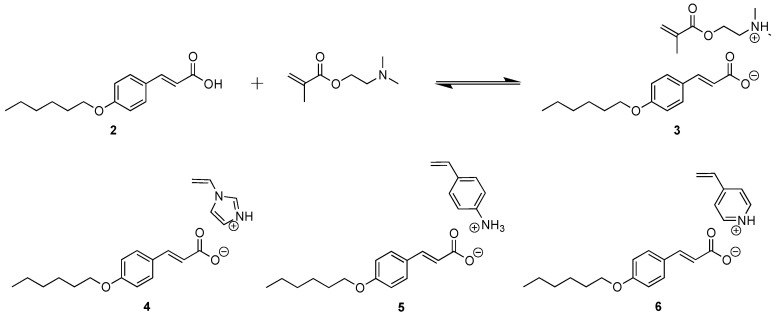
Synthesis of 2-(dimethyl ammonium)ethyl methacrylate p-hexoxy coumarate 3, [DMAEM+ HexOCou-]; Chemical structures of 4-vinylimidazolium p-hexoxy coumarate 4, [VIm+ HexOCou-], 4-vinylanilinium p-hexoxy coumarate 5, [VAn+ HexOCou-] and 4-vinylpyridinium p-hexoxy coumarate 6, [VPy+ HexCou-].

2-(dimethyl amino)ethyl methacrylate and p-hexoxy coumarate 2 were weighted and mixed in an equimolar amount. The product was obtained instantly as a viscous liquid (Scheme 2).

2-(dimethyl ammonium)ethyl methacrylate p-hexoxy coumarate 3. Yield 100%; ^1^H NMR (400 MHz, D_2_O) δ (ppm): 1.04 (t, 3 H, CH_3_), 1.36 (m, 4 H, CH_3_(CH_2_)_2_), 1.48 (m, 2 H, O(CH_2_)_2_CH_2_), 1.92 (m, 2 H, OCH_2_CH_2_), 2.08 (s, 3 H, CCH_3_), 2.58 (6 H, N^+^H(CH_3_)_2_), 2.98 (t, 2 H, N^+^HCH_2_), 4,10 (t, 2 H, OCH_2_), 4.48 (t, 2 H, OCH_2_), 5.72 (s, 1 H, C=CH_2_), 6.28 (s, 1 H, C=CH_2_), 6.44 (d, 1 H, CH=CHCOO), 7.00 (d, 2 H, Ar–H), 7.58 (d, 2 H, Ar–H), 7.66 (d, 1 H, CH=CHCOO); FTIR (resolution of 2 cm^−1^) (cm^−1^): 3300 (N–H), 2970 (C–H), 1719 (C=O), 1635 (C=C), 830 (H–C=C)

#### 2.2.3. Synthesis of 4-Vinylimidazolium p-Hexoxy Coumarate 4, [VIm+ HexOCou-]

1-vinylimidazole and p-hexoxy coumaric acid were weighted and mixed in an equimolar amount. The product was obtained instantly as a viscous liquid. 1-vinylimidazium p-hexoxy coumarate 4. Yield 100%; ^1^H NMR (400 MHz, D_2_O) δ (ppm): 1.18 (t, 3 H, CH_3_), 1.31 (m, 4 H, CH_3_(CH_2_)_2_), 1.41 (m, 2 H, O(CH_2_)_2_CH_2_), 1.78 (m, 2 H, OCH_2_CH_2_), 4.12 (t, 2 H, OCH_2_), 5.00 (d, 1 H, C=CH_2_), 5.44 (d, 1 H, C=CH_2_), 6.91 (d, 1 H, CH=CHCOO), 7.09 (m, 2 H, N+HCH=CHN), 7.33 (d, 2 H, Ar–H), 7.45 (s, 1 H, NCH=C), 7.52 (d, 2 H, Ar–H), 7.54 (d, 1 H, CH=CHCOO), 7.89 (s, 1 H, N+H=CHN). FTIR (resolution of 2 cm^−1^) λ (cm^−1^): 3340 (N–H), 2930 (C–H), 1675 (C=O), 1620 (C=C), 830 (H–C=C)

#### 2.2.4. Synthesis of 4-Vinylanilinium p-Hexoxy Coumarate 5, [VAn+ HexOCou-]

4-vinylaniline and p-hexoxy coumaric acid were weighted and mixed in an equimolar amount. The product was obtained instantly as a viscous liquid. 4-vinylanilinium p-hexoxy coumarate 5 (Scheme 2). Yield 100%; ^1^H NMR (400 MHz, D_2_O) δ (ppm): 1.18 (t, 3 H, CH_3_), 1.33 (m, 4 H, CH_3_(CH_2_)_2_), 1.46 (m, 2 H, O(CH_2_)2CH_2_), 1.78 (m, 2 H, OCH_2_CH_2_), 4.12 (t, 2 H, OCH_2_), 5.11 (d, 1 H, C=CH_2_), 5.64 (d, 1 H, C=CH_2_), 6.70 (d, 1 H, CH=CHCOO), 6.82 (d, 2 H, Ar–H), 6.91 (d, 1 H, CH=C), 7.02 (d, 2 H, (N^+^H_3_)Ar–H), 7.34 (d, 2 H, Ar–H), 7.52 (d, 2 H, Ar–H), 7.57 (d, 1 H, CH=CHCOO). FTIR (resolution of 2 cm^−1^) λ (cm^−1^): 3360 (N–H), 2930 (C–H), 1675 (C=O), 1620 (C=C), 829 (H–C=C)

#### 2.2.5. Synthesis of 4-Vinylpyridinium p-Hexoxy Coumarate 6, [VPy+ HexCou-]

4-Vinylpyridine and p-hexoxy coumaric acid were weighted and mixed in an equimolar amount. The product was obtained instantly as a viscous liquid. 4-vinylpyridinium p-hexoxy coumarate 6 (Scheme 2). Yield 100%; ^1^H NMR (400 MHz, D_2_O) δ (ppm): 1.18 (t, 3 H, CH_3_), 1.41 (m, 4 H, CH_3_(CH_2_)_2_), 1.46 (m, 2 H, O(CH_2_)_2_CH_2_), 1.77 (m, 2 H, OCH_2_CH_2_), 4.10 (t, 2 H, OCH_2_), 5.57 (d, 1 H, C=CH_2_), 6.09 (d, 1 H, C=CH_2_), 6.77 (d, 1 H, CH=CHCOO), 7.00 (d, 2 H, Ar–H), 7.48 (d, 2 H, Ar–H), 7.52 (d, 2 H, Ar–H), 7.56 (d, 2 H, Ar–H), 8.45 (d, 2 H, Ar–H). FTIR (resolution of 2 cm^−1^) λ (cm^−1^): 3350 (N–H), 2940 (C–H), 1683 (C=O), 1635 (C=C), 830 (H–C=C)

#### 2.2.6. Preparation of the UV-Polymer Coatings

A typical acrylic UV-Curable coating formulation is composed of Oxybis(propane-1,2-diyl) diacrylate (40 wt%), dipropylene glycol diacrylate (25 wt%), trimetyl propyl triacrylate (13 wt%), cyclic trimethylolpropane formal acrylate (12 wt%), acid based adhesion promotor (3 wt%), Darocur (Speedcure 73) (5 wt%) and a coumarate monomer (20 wt%) were added and mixed in a vial.

Mild Steel AS1020 surface were degreased with acetone at ambient temperature. Before casting the monomer solution on the substrate, a rectangular gap (3 × 5 cm) of a depth of 62.5 μm was created using tape. Then, the gap was filled with the aforementioned monomer mixture and it was UV cured in 120 s, using a UVC-5 (DYMAX) UV Curing Conveyor System with an intensity up to 400 mW/cm^2^, 30 mm lamp-to-belt distance, and belt speed at 7 m/min.

### 2.3. Characterization Methods

#### 2.3.1. Nuclear Magnetic Resonance (NMR)

NMR spectra were recorded on a Bruker AC-400 (^1^H-NMR) spectrometer ((Bruker, Billerica, MA, USA ) with the following experimental conditions: spectral width 15 ppm with 32 k data points, flip angle 908, relaxation delay of 1 s, digital resolution of 0.24 Hz/pt.

#### 2.3.2. Attenuated Total Reflectance–Fourier Transport Infrared Spectroscopy (ATR-FTIR)

Attenuated total reflection–Fourier transform infrared spectroscopy (ATR-FTIR) measurements were performed on Bruker Alpha-P equipment (Bruker, Billerica, MA, USA).The spectra were recorded from 50 to 4000 cm^−1^ with a resolution of 2 cm^−1^.

#### 2.3.3. Potentiodynamic Polarization (PP) Experiments

A BioLogic VMP3 multi-channel potentiostat (Biologic, Seyssinet-Pariset, France) combined with EC Lab V10.44 software were used for PP experiments. A three-electrode cell was used with the steel rod as the working electrode, a titanium mesh counter electrode and Ag/AgCl reference electrode. The reference electrode was placed in a Luggin capillary that was positioned close to the working electrode surface. The open circuit voltage (OCV) was monitored for 30 min followed by the PP scan at a rate of 0.167 mV/s, with a scan range of 150 below to 250 mV above OCV. Three PP curves were obtained for each test solution.

- Corrosion current density (i_corr_) and corrosion potential (E_corr_)

Specific i_corr_ and E_corr_ values were extracted from the PP curves using Tafel extrapolation. A value for i_corr_ was taken as the point where the linear section of the anodic and cathodic sections of the PP curves intersected the value for E_corr_.

From the i_corr_ values, inhibitor efficiencies (IE) were calculated according to Equation (1):

(1) $$\mathrm{IE}=\;\frac{i_{\mathrm{corr}\;\mathrm{control}}-{\;i}_{\mathrm{corr}\;\mathrm{inhibited}}}{i_{\mathrm{corr}\;\mathrm{control}}}\times 100$$

The electrochemical impedance spectroscopy (EIS), over a test period of 24 h, was carried out in order to characterize the electrochemical properties of AS1020 mild steel electrodes immersed in the control and inhibited solution. The aforementioned BioLogic VMP3 multi-channel potentiostat was used for the EIS tests. The OCV was monitored over a frequency range from 100 kHz to 10 mHz with 6 points per decade and a sinusoidal amplitude of 10 mV. Impedance responses were monitored after each hour.

#### 2.3.4. Immersion Test

Immersion tests were carried out in order to observe and compare the inhibition performance of the different inhibitor compounds. Thereby, 24-h immersion tests of mild steel AS1020 in 0.01 M NaCl aqueous solution with and without 8 mM of inhibitor monomers were carried out. Surfaces of mild steel, after immersion, were rinsed with MiliQ water, dried with N_2_ gas, and dried in a dessicator for 2 h.

#### 2.3.5. Scanning Electron Microscopy (SEM) and Energy-Dispersive X-Ray Spectroscopy (EDS)

SEM and EDS were used to observe mild steel surfaces after the immersion test. A JSM-IT300 LV SEM instrument (JEOL, Tokyo, Japan), with an Oxford instrument X-Max 50 mm^2^ EDS detector at 15 kV, was used with an accelerating voltage of 20 kV. EDS spectra collected for 60 s were produced using AZtec software.

#### 2.3.6. Scribe Test

A scalpel blade was used to make a 10 mm artificial defect within the polymer coating at the center of each coated surface. The filiform test was adopted from DIN 65472 (Deutsches Institut für Normung, Berlin, Germany). Scribed samples were placed over a small beaker containing 20 mL of concentrated HCl acid (32%) for 15 min in order to activate corrosion. After activation, samples were placed into a plastic container containing a beaker of saturated KCl. All the samples were left for 10 days in an oven at 45 ± 2 °C to obtain a relative humidity of 85% [2].

#### 2.3.7. Water Uptake of the Polymer Coatings

Polymer films were weighed at time zero and subsequently immersed in MiliQ water. After immersion times of 24, 48, and 72 h, the polymer films were dried with a tissue and weighed in order to evaluate its water uptake or weight loss.

## 3. Results and Discussion

### 3.1. Mild Steel Corrosion Inhibition Properties of p-Hexoxy Coumarate Based Monomeric Ionic Liquids

**Figure 1:**
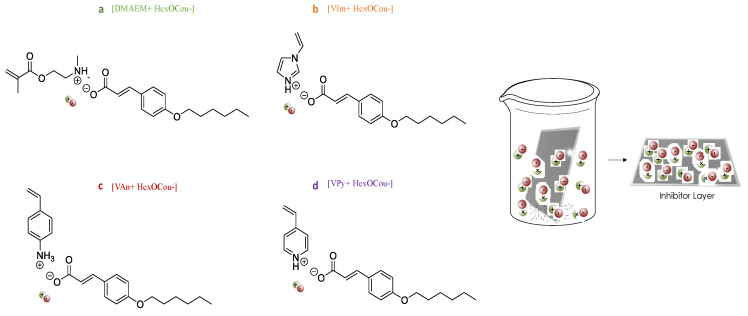
Chemical structures: (**a**) 2-(Dimethylammonium)ethyl methacrylate hexoxy p-coumarate ([DMAEM+ HexOCou-]), (**b**) 1-vinylimidazolium p-hexoxy coumarate ([VIm+ HexOCou-]), (**c**) 4-vinylanilinium p-hexoxy coumarate ([VAn+ HexOCou-]) and (**d**) 4-Vinylpyridinium p-hexoxy coumarate ([VPy+ HexOCou-]); Representation of an immersion of mild steel 1020 surface in an aqueous solution containing the ionic liquid monomer inhibitors.

In order to investigate the effect of the cation on the corrosion inhibition properties of the coumarate ionic liquids, we selected four different common cationic monomers having ammonium, imidazolium, pyridinium and anilinium cations. The protic ionic liquid monomers were easily obtained by acid-base proton exchange reaction between commercially available monomers and p-hexoxy coumaric acid as described in Scheme 2 shown in the experimental section. The chemical structure of the four monomers investigated in this work is shown in Figure 1, [DMAEM+ HexOCou-] [VIm+ HexOCou-] [VAn+ HexOCou-] and [VPy+ HexCou-]. First, the corrosion inhibition properties of the monomers were evaluated by immersing mild steel AS1020 foils into an aqueous solution of the ionic monomers. By this method, it is expected that the organic ionic compounds may adsorb onto the mild steel surface forming a corrosion inhibition layer, as illustrated in the Figure 1.

**Figure 2:**
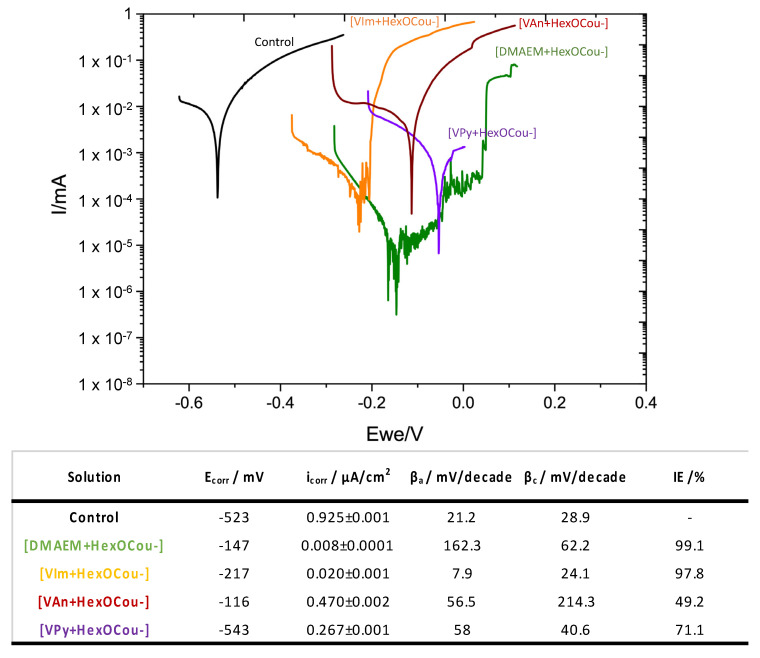
Potentiodynamic polarization curves of AS1020 mild steel after 24 h at OCV in control and 8 mM aqueous solutions of the monomeric ionic liquids; Corrosion potentials (E_corr_), corrosion current density (i_corr_), tafel anodic and cathodic slopes (β_a_ and β_c_) of the control and inhibitor samples.

Potentiodynamic polarization scans of AS1020 mild steel after an exposure of 24 h in 0.01 M NaCl control solution and inhibitors containing solutions (0.01 M NaCl + 8 mM inhibitor monomers) are shown in Figure 2. Corrosion potentials (E_corr_), corrosion current density (i_corr_), tafel anodic and cathodic slopes (β_a_ and β_c_) calculated using Tafel extrapolation are displayed in Figure 2. It can be observed that, for all the inhibitors, the corrosion potential (E_corr_) is shifted towards a more positive value compared with the control. The E_corr_ of the control is at −523 mV whereas the inhibitors present potentials ranging from −116 to −543 mV, meaning that all the inhibitors are mainly affecting and suppressing the anodic reaction of the corrosion. The potentiodynamic polarization curves indicate that all compounds acted as anodic inhibitors. The addition of inhibitors to the control solution shifted the corrosion potentials (E_corr_) to more positive values and significantly reduced the anodic current density [13,26,27]. Anodic inhibitors block the anodic reaction (oxidation of Fe to Fe^2+^) by a creation of a barrier coating on anodic sites. They are adsorbed on the metal surface forming a protective film, so reducing the corrosion current and increasing the corrosion potential [13,26,27,28,29,30]. However, differences in both the anodic and cathodic Tafel slopes can be observed, suggesting that, apart from blocking the anodic reaction, the cathodic reaction is also affected by the presence of these compounds. Moreover, the potentiodynamic polarization results also show significant current fluctuations. Those fluctuations refer with metastable pitting, in which there is a constant breakdown and repair of a passive film [1,2]. In most cases, the corrosion current decreased considerably compared with the control, yielding very high inhibitor efficiency values. [DMAEM+ HexOCou-]), showed a corrosion current density (i_corr_) of 0.008 μA/cm^2^ and an inhibitor efficiency of 99.1%. Finally, the vinylic compounds, [VIm+ HexOCou-], [VAn+ HexOCou-], and [VPy+ HexOCou-], produced corrosion current densities (i_corr_) of 0.020, 0.470, and 0.267 μA/cm^2^ and acceptable inhibitor efficiencies of 97.8%, 49.2% and 71.1%, respectively. A dramatic effect of the cationic component of the ionic liquid in the corrosion inhibition process is observed. Among the different monomers the inhibition efficiency was [DMAEM+ HexOCou-] > [VIm+ HexOCou-] > [VPy+ HexCou-] >> [VAn+ HexOCou-]. So, the best results were obtained for the compound containing ammonium derivative cation, followed by the one containing imidazolium and pyridinium cations. Finally, the inhibitors containing anilinium cation showed i_corr_ almost unchanged relative to the control even though E_corr_ was shifted considerably.

**Figure 3:**
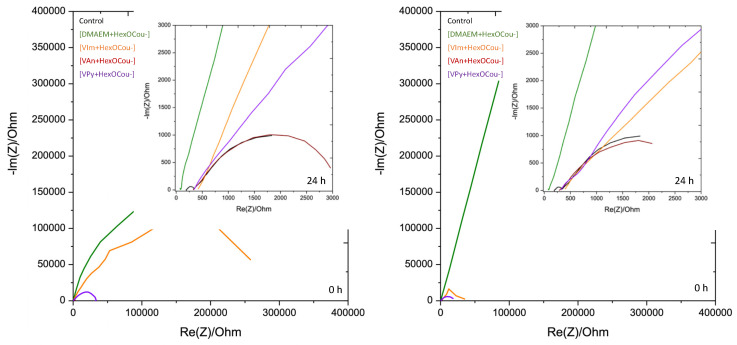
Nyquist spectra for AS1020 mild steel immersed in the control and the inhibited solutions at 0 h and 24 h.

Electrochemical Impedance Spectroscopy experiments were carried out in order to further characterize the anticorrosive capacity of different inhibitors. The impedance responses were measured during an immersion in NaCl 0.01 M aqueous solution for 24 h. Figure 3 shows the Nyquist plot at the beginning of the test and after 24 h of immersion in NaCl 0.01 M aqueous solution. The [DMAEM+ HexOCou-] inhibitor presents the largest impedance consistent with the PP data above and reflecting a stronger interaction between the inhibitor and mild steel surface. Moreover, it can be observed that after 24 h of immersion the anticorrosive capacity of [DMAEM+ HexOCou-] inhibitor is not decreased. On the other hand, varying the structure of the inhibitors, a difference in the magnitude of the impedance can be seen depending on the cationic part. [VIm+ HexOCou-] and [VPy+ HexOCou-] inhibitors present a bigger capacitive loop than the control although significantly smaller than for the DMAEM compound. In the case of the [VAn+ HexOCou-] inhibitor, the initial EIS meaurement is similar to the control, but at the end of the 24 h experiment, the impedance is even a little lower than the control suggesting poorer corrosion resistance of the mild steel under these conditions. This data is fully consistent with the PP data above and suggests a. co-dependence of the anion and cation on the adsorption on steel from aqueous solution and hence a significant effect on the corrosion performance.

**Figure 4:**
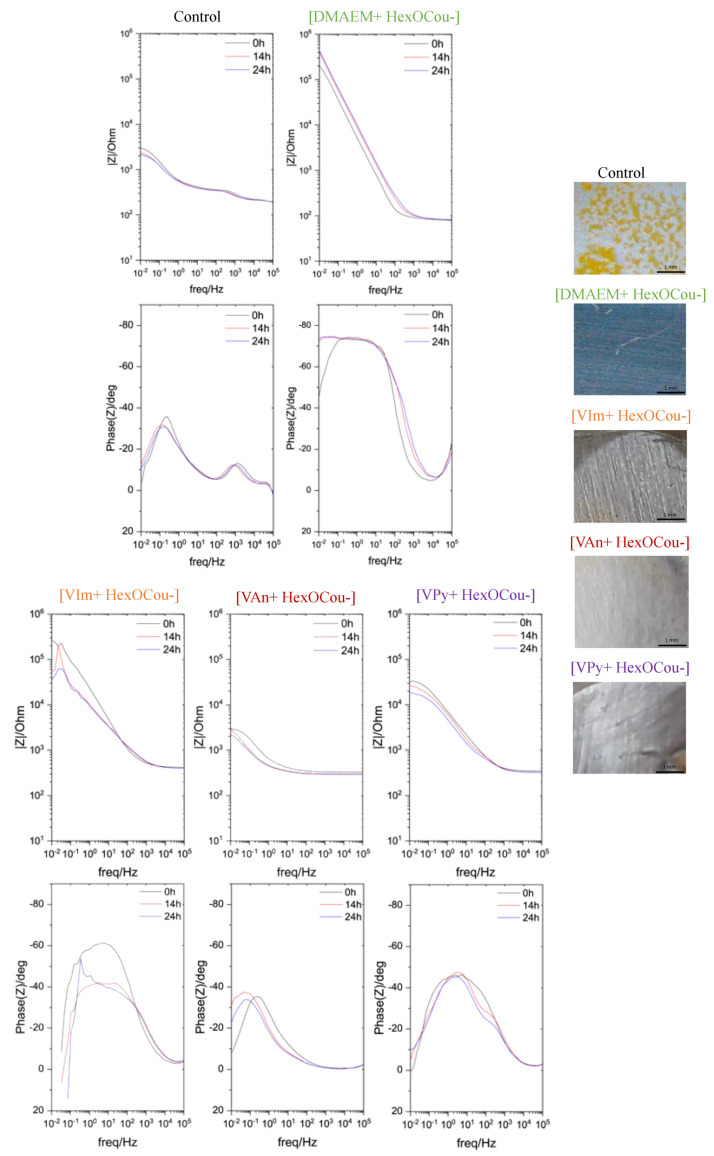
Optical microscopy images and electrochemical impedance spectra for AS1020 mild steel immersed in the control and the inhibited solutions up to 24 h: impedance modulus and phase angle plots for control; inhibited solution containing [DMAEM+ HexOCou-]; inhibited solution containing [VIm+ HexOCou-]; inhibited solution containing [VAn+ HexOCou-]; inhibited solution containing [VPy+ HexOCou-].

The Bode plots of the control and the comparison with the different inhibitors as a function of immersion time are shown in the Figure 4. Due to corrosion reaction occurring on the control sample, in the low frequency range, a decrease in the impedance and the phase angle plateau can be observed, passing from 10^3.48^ Ωcm^2^ and 35° at 2 h of immersion to 10^3.34^ Ωcm^2^ and 30° at 24 h of immersion. On the other hand, steel surfaces immersed in the inhibitors shows different anticorrosive responses. Specifically, the mild steel sample which was immersed in the ammonium coumarate [DMAEM+ HexOCou-] monomer presents an increase on the impedance obtaining 10^5.64^ Ωcm^2^ at the low frequency range. In the corresponding phase angle of the time constant a plateau is observed at 74°, showing that the inhibitor results in predominantly capacitive behavior and correlates with the improved anticorrosive performance. The imidazolium, anilinium, and pyridinium monomers were also compared using the Bode formalism as discussed below.

Mild steel, after an immersion of 2 h in the imidazolium monomer [VIm+ HexOCou-] solution, presented an impedance of 10^5.42^ Ωcm^2^, although this value decreased to 10^4.54^ Ωcm^2^ after 24 h. The corresponding phase angle at the low frequency range also decreased with time from 61° to 53°. It is worth noting that, upon initial exposure, this inhibitor blocks the corrosion reaction almost as effectively as the DMAEM inhibitor, although the corrosion inhibition effectiveness was lost over time. On the other hand, the vinyl anilinium based monomer shows no effectiveness as an inhibitor which is very surprising given the fact that the anion is common and is expected to be the inhibiting moiety. The Bode plots for the pyridinium monomer show an impedance of 10^4.50^ Ωcm^2^ after the first 2 h whereas after 24 h this value is also decreased to 10^4.26^ Ωcm^2^ with a phase angle of 45°. Optical microscopy images were taken in order to correlate with the EIS results. Figure 4 shows optical images of the samples immersed in control, [DMAEM+ HexOCou-], [VIm+ HexOCou-], [VAn+ HexOCou-], and [VPy+ HexOCou-]. The control sample presents a surface covered by red rust, while the inhibitor immersed samples are not showing evidence of such corroded areas.

**Figure 5:**
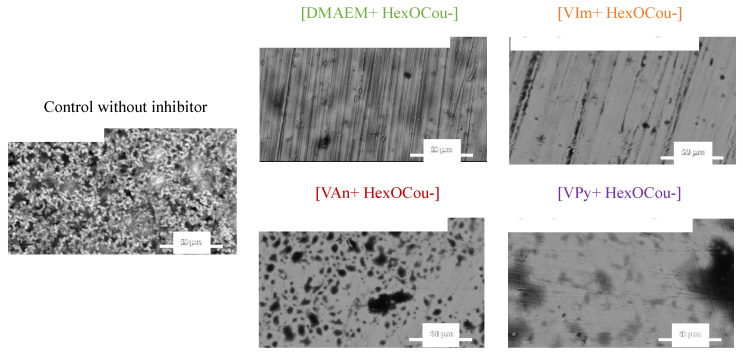
SEM images of AS1020 mild steel after an exposure of 24 h in 0.01M NaCl control solution and inhibitors containing solutions (0.01 M NaCl + 8 mM inhibitor monomers).

In order to corroborate this trend and the corrosion inhibition effect we evaluated all the samples using electron microscopy of the surfaces. Mild steel AS1020 surfaces were analyzed after an immersion of 24 h in 0.01 M NaCl with and without monomeric ionic liquid inhibitors by optical microscopy, scanning electron microscopy and electron diffraction spectroscopy. In Figure 5, rust deposits can be observed on the surface immersed in the control solution (optical and scanning electron microscopy images). The EDS data (Supplementary Figure S1) confirms that those precipitates are mainly iron oxide. As it can be seen in the optical images of all samples, the surfaces in contact with solutions containing inhibitors do not present rust deposits although pitting is still evident to different extents in these inhibited samples. EDS analysis confirmed the presence of carbon, oxygen, and nitrogen atoms on these surfaces, indicating the creation of an organic inhibiting layer onto the metallic surface. The surfaces exposed to DMAEM and VIm hexoxy coumarate show the least corrosive attack, once again consistent with the electrochemistry presented above. Thus, it can be concluded that both the p-hexoxy coumarate anion and the cationic monomer have an important effect in the corrosion inhibition phenomena.

### 3.2. Covalent Incorporation of p-Hexoxy Coumarate Ionic Liquid Monomers into Acrylic Polymer Coatings by UV-Photopolymerization

**Scheme 3:**
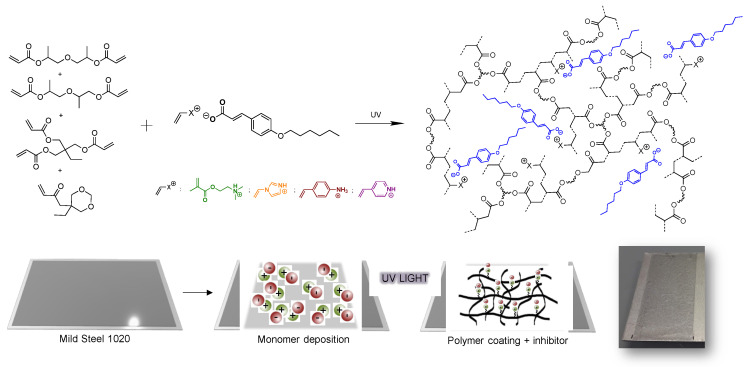
Chemical structures of monomers and polymer coatings obtained by UV-photopolymerization on mild steel 1020 surface.

Photopolymerization was carried out in order to form polymer coatings onto the mild steel AS1020 surface (Scheme 3). To a typical UV-curable acrylic formulation including a mixture of mono and difunctional acrylic monomers and a photoinitiator, 20 wt% of the different coumarate monomers were added. After UV radiation, the liquid resin became a solid coating and the polymerization of the acrylic double bonds was confirmed by ATR-FTIR. Supplementary Figure S2 in the supplementary material shows ATR-FTIR spectra of all four coatings comparing the control coating, the monomer mixture and the final coating containing 20 wt% coumarate ionic units. In all the coatings, the disappearance of the band between 1600–1650 cm associated to the double bond can be observed, which confirms the high extent of the photopolymerization (>90% in all the cases). By this method, the cationic moiety of the coumarate ionic liquid monomer is covalently attached to the acrylic network whereas the coumarate anion is interacting ionically with the polymer backbone.

First, we investigated the stability of polymer coatings towards water uptake and leaching of the coumarate counter-anion. It is worth mentioning that, leaching out of the coatings of the corrosion inhibitors when added as additives is one of the main limitations. Whereas in this paper we proposed to attach part of the corrosion inhibitor covalently to the coating by copolymerization and to retain the other active ingredient through strong coulombic interactions. The polymer coatings were immersed in water for 24, 48 and 72 h, patted dry and then weighed so as to study the differences in mass uptake or loss (Supplementary Figure S3). Interestingly, the acrylic coatings are not absorbing water in spite of the presence of the hydrophilic ionic monomers. Leaching from the film of the unreacted monomer or pendant counter-anions can thus be analyzed for each coating. Interestingly, the Acrylic UV-coatings did not show a significant reduction in mass indicating that the coumarate inhibitors are well integrated into the acrylic polymer coating. Only, the [VPy+ HexOCou-] based coating lost 4% of its mass in the first 24 h and 8% after 72 h which indicates less integration in the polymer coating.

**Figure 6:**
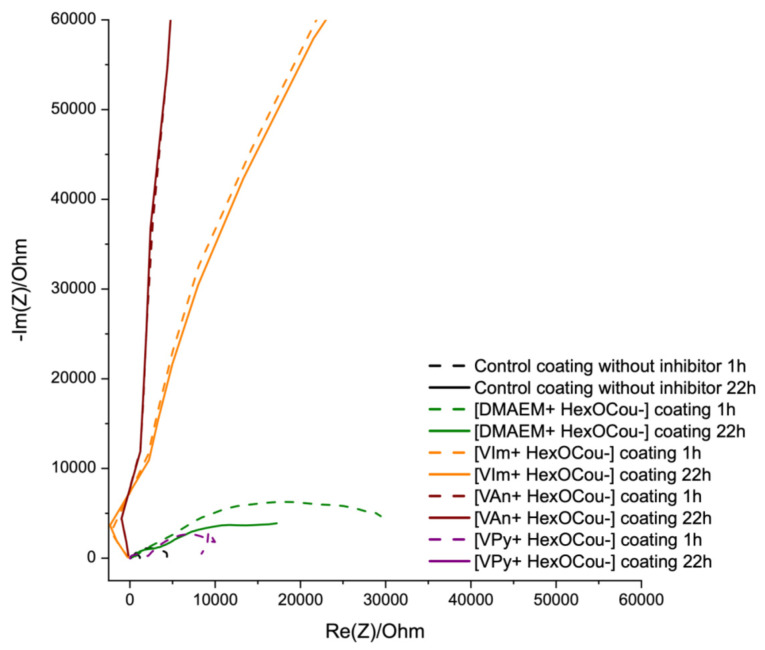
Nyquist plot of polymer coating on AS1020 mild steel containing 20% of [DMAEM+ HexOCou-] (green), 20% of [VIm+ HexOCou-] (orange), 20% of [VAn+ HexOCou-] (wine), 20% of [VPy+ HexOCou-] (purple) and control (black) immersed in 0.005 M NaCl after 22 h.

The anticorrosion profile of each polymer coating containing coumarate inhibitors was tested by EIS measurements. Figure 6 shows the Nyquist plot of different polymer coatings after an immersion of 22 h in NaCl 0.005 M aqueous solution (Supplementary Figure S4). All the coated samples including ionic monomers showed a bigger depressed semicircle than the semicircle of the UV-cured coating control without ionic monomer. The samples coated with acrylic formulations having 20 wt% of [VIm+ HexOCou-] and [VAn+ HexOCou-] showed the biggest capacitive loop that is constant during the length of the experiment. The [VPy+ HexOCou-] containing coating showed less anticorrosive performance amongst the different coatings containing inhibitor which also correlates with its poor performance as a monomer inhibitor.

**Figure 7:**
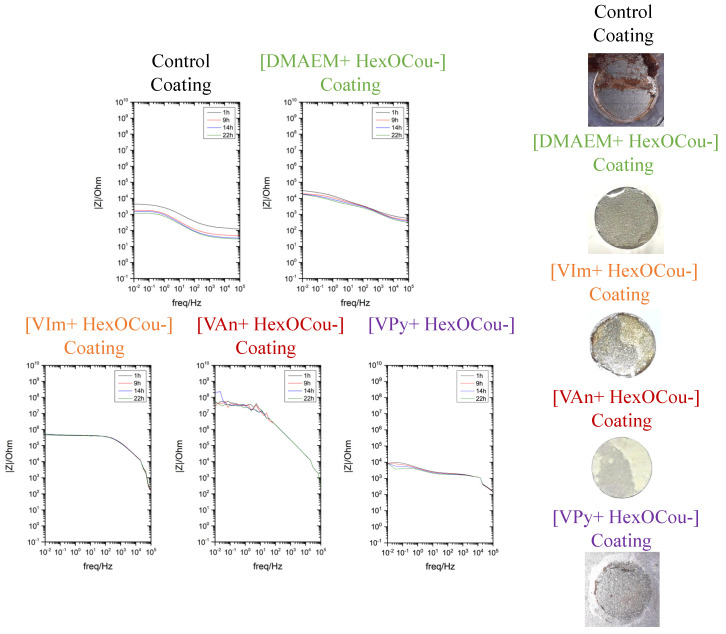
Optical microscopy images of polymer coatings on AS1020 mild steel containing 20% of [DMAEM+ HexOCou-], 20% of [VIm+ HexOCou-], 20% of [VAn+ HexOCou-], 20% of [VPy+ HexOCou-] and control immersed in 0.005 M NaCl after 20h; Electrochemical impedance spectra of different polymer coatings on AS1020 mild steel immersed in 0.005 M NaCl at different immersion times: impedance modulus plots for control, inhibited coating containing 20% [DMAEM+ HexOCou-]; inhibited coating containing 20% [VIm+ HexOCou-]; inhibited coating containing 20% [VAn+ HexOCou-]; inhibited coating containing 20% [VPy+ HexOCou-].

Pictures of all coatings were taken after EIS measurements (Figure 7). As aforementioned, the control coating presents some rust deposits on the surface. In contrast, [DMAEM+ HexOCou-], [VIm+ HexOCou-], and [VAn+ HexOCou-] based polymer coatings do not suffer any deterioration. However, as the Nyquist plot showed, the [VPy+ HexOCou-] based coating presents a corroded site which leads to a decrease in the impedance of the coating and thus a decrease in anticorrosion activity (Figure 6). Figure 7 shows the magnitude of the impedance responses in the form of the Bode plot for [DMAEM+ HexOCou-], [VIm+ HexOCou-], [VAn+ HexOCou-], and [VPy+ HexOCou-] based polymer coatings. The initiation of corrosion can be observed in the control coating due to a decrease in the impedance and in the plateau of the phase angle in the low frequency range (10^−1^–10^1^) with time. The Bode plots for the [DMAEM+ HexOCou-] based coating show an impedance of 10^4.47^ Ωcm^2^ at the low frequency range after 1 h of immersion in NaCl 0.005 M aqueous solution. This impedance is reduced to 10^4.24^ Ωcm^2^ after 9 h and it remains constant until 22 h. That variation corresponds to the initiation of corrosion and the gradual formation of the corrosion products therefore only providing a limited level of protection against mild steel corrosion relative to control. On the other hand, polymer coatings based on [VIm+ HexOCou-] and [VAn+ HexOCou-] present the highest impedance values, which are also constant in time, 10^5.70^ Ωcm^2^ and 10^7.70^ Ωcm^2^, respectively. Accordingly, they present a high phase angle value, 78° and 85°, respectively (Supplementary Figure S5). The polymer coating with 20% [VAn+ HexOCou-] showed the best anticorrosive profile, in fact, due to the high impedance and phase angle data and the sensitivity of the experiment, noisy data can be observed. As observed from the Nyquist plot, the [VPy+ HexOCou-] based coating showed lower impedance during immersion. In fact, as the leaching test on [VPy+ HexOCou-] based coating showed, the concentration of the inhibitor in the polymer may be reduced, and as the Bode plot demonstrates, the anticorrosive effect is partly lost.

#### Scribe Test

**Figure 8:**
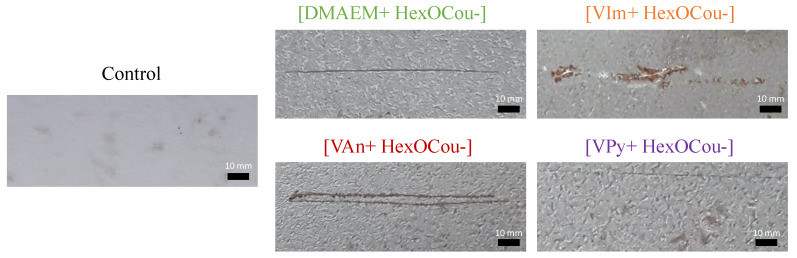
Scribe test images of control coating and coatings based on different inhibitors.

The scribe test was carried out onto in order to further compare the different coatings. A defect was introduced into a reference UV polymer coating without any inhibitor, and coatings containing 20 wt% [DMAEM+ HexOCou-], [VIm+ HexOCou-], [VAn+ HexOCou-], and [VPy+ HexOCou-] respectively. The coatings were introduced into acidic solutions in order to initiate the corrosion reaction. In Figure 8, filiform corrosion pictures of all coatings can be observed after 10 days. As can be observed, the control coating without coumarate inhibitors showed a completely rust covered surface. On the other hand, the polymer coatings containing inhibitors passed very well the scribe test showing very little corrosion propagation. As shown in the pictures the polymer coatings including [DMAEM+ HexOCou-], [VIm+ HexOCou-], and [VAn+ HexOCou-] showed excellent performance. On the other hand, the coating including [VPy+ HexOCou-] showed the worst corrosion inhibition. These data are only qualitative, but still it is interesting to note that the order of performance from this test is different to the EIS data discussed above. These differences may be related to the different initiation mechanism for corrosion, (i.e., acid exposure in the scribe test versus chloride in the immersion tests).

## 4. Conclusions

In this article, the effect of the cation in the corrosion inhibition properties of coumarate based ionic liquid monomers and UV-coatings were investigated. First, potentiodynamic polarization, electrochemical impedance spectroscopy experiments, and surface analyses were undertaken in order to verify the corrosion inhibition performance of the monomers on the mild steel AS1020 surface. The most promising inhibitor monomer in solution is [DMAEM+ HexOCou-], showing an inhibition efficiency of 99.1% in solution following the order [DMAEM+ HexOCou-] > [VIm+ HexOCou-] > [VPy+ HexCou-] >> [VAn+ HexOCou-]. In fact, at the concentrations tested, the [VAn+ HexCou-] compound slightly accelerated corrosion, whereas the other compounds can be considered as excellent inhibitors.

The ionic liquid monomers were covalently incorporated into an acrylic coating formulation by UV photopolymerization. The polymer UV-coatings did not show a significant reduction in mass indicating that the coumarate inhibitor are well integrated into the acrylic polymer coating using this strategy. In this case, the polymer coating with the highest anti-corrosive properties is the one containing [VIm+ HexOCou-] and [VAn+ HexOCou-] followed by [DMAEM+ HexOCou-], as determined from the EIS measurements and scribe tests. It is worth noting that this behavior is slightly different than that observed when the monomers were dissolved and evaluated as corrosion inhibitors in aqueous solutions. Although the reasons for these differences are not clear as yet, we would like to emphasize that the experimental conditions (monomer vs polymer, and aqueous solution, vs. polymer coatings) are quite different in the two cases. It seems that the π-electrons of the Vim and VAn aromatic rings can interact with the metal vacant d-orbital and show a better adsorption and protection against corrosion, particularly in the polymer coatings.

Overall, we can conclude that the cationic moiety of the monomers and its interaction with the hexoxy coumarate anion play a role in the mechanism of adsorption onto the mild steel surface and its subsequent ability to inhibit corrosion. We also demonstrate that the covalent incorporation of the coumarate ionic liquids into acrylic UV coatings is a successful strategy. Interestingly, these polymer coatings combined the beneficial barrier properties and the effect of the coumarate ionic liquids as corrosion inhibitors.

## Supplementary Materials

The following are available online at https://www.mdpi.com/2073-4360/12/11/2611/s1, Figure S1: EDS data of 3 different zones (1: mild steel surface; 2: Rust deposits; 3: inhibitor deposits) bare mild steel and mild steel covered with inhibitor after 24 h immersed in 0.01 M NaCl, Figure S2: ATR-FTIR of inhibitors based monomer mixture (c) and coatings (b) compared to the control coating (a). I: [p-O(C_6_H_13_)coum] MA; II: [p-O(C_6_H_13_)coum] IM; III: [p-O(C_6_H_13_)coum] AN; IV: [p-O(C_6_H_13_)coum] PY, Figure S3: Swelling test of polymer coatings. Weight of polymers after 24, 48 and 72 h immersion in water, Figure S4: Nyquist plot of polymer coating on AS1020 mild steel containing 20% of [DMAEM+ HexOCou-] (green), 20% of [VIm+ HexOCou-] (orange), 20% of [VAn+ HexOCou-] (wine), 20% of [VPy+ HexOCou-] (purple) and control (black) immersed in 0.005M NaCl after 22h, Figure S5: Electrochemical impedance spectra of different polymer coatings on AS1020 mild steel immersed in 0.005M NaCl at different immersion times: phase angle plots for control, inhibited coating containing 20% [DMAEM+ HexOCou-]; inhibited coating containing 20% [VIm+ HexOCou-]; inhibited coating containing 20% [VAn+ HexOCou-]; inhibited coating containing 20% [VPy+ HexOCou-].
