# Supplementary material for: Cation Effect in the Corrosion Inhibition Properties of Coumarate Ionic Liquids and Acrylic UV-Coatings
Source: Polymers (Basel). 2020 Nov 6;12(11):2611. doi: 10.3390/polym12112611 (PMC7694658; doi:10.3390/polym12112611)
Supplement: Supplementary file 1 [file polymers-12-02611-s001.pdf]

polymers

MDPI

Article

Cation Effect in the Corrosion Inhibition Properties of Coumarate based poly(ionic liquid)s

Esther Udabe [1], Anthony Somers [2], Maria Forsyth[1,2,3,*] and David Mecerreyes [1,3,*]

[1] POLYMAT University of the Basque Country UPV/EHU, Donostia-San Sebastian 20018, Spain;
[2] Institute for Frontier Materials, Deakin University, Geelong, Victoria, 3220, Australia,
[3] IKERBASQUE Basque Foundation for Science, Bilbao, Spain
* Correspondence: (M.F.) maria.forsyth@deakin.edu.au; (D.M) david.mecerreues@ehu.es

Received: date; Accepted: date; Published: date

	1	2	3
	Wt %		
Fe	56,5	88,8	37,9
O	34,2	5,5	5,8
C	8,4	4,1	47,7
N	-	-	6,8

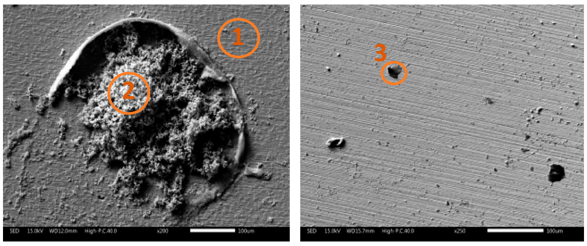

Figure S1. EDS data of 3 different zones (1: mild steel surface; 2: Rust deposits; 3: inhibitor deposits) bare mild steel and mild steel covered with inhibitor after 24h immersed in 0,01M NaCl.

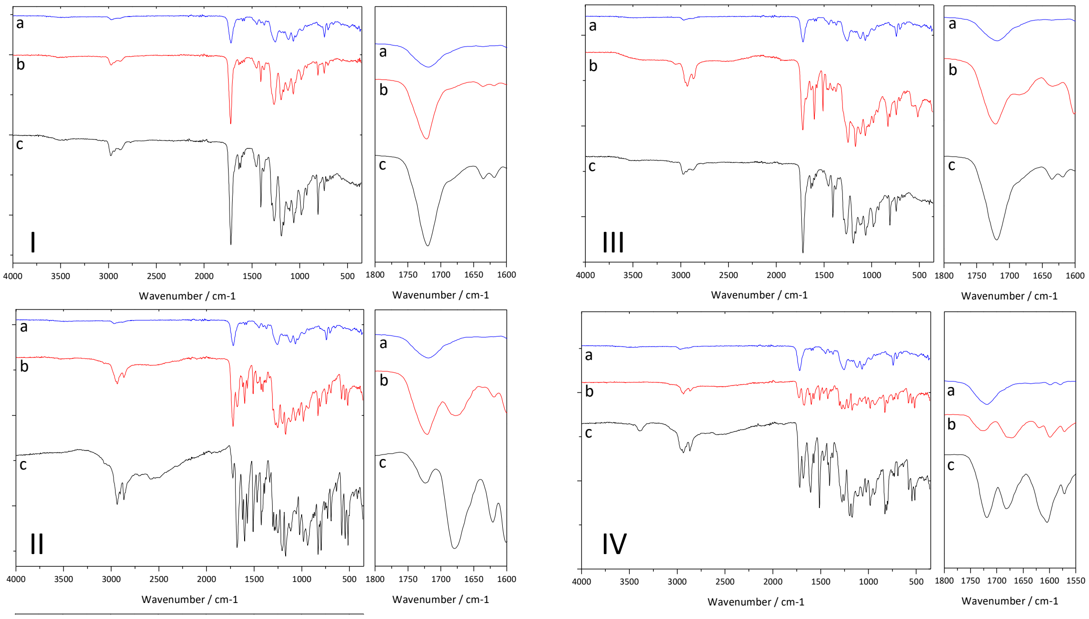

Figure S2. ATR-FTIR of inhibitors based monomer mixture (c) and coatings (b) compared to the control coating (a). I: [DMAEM+ HexOCou-]; II: [VIm+ HexOCou]; III: [VAn+ HexOCou-]; IV: [VPy+ HexOCou-].

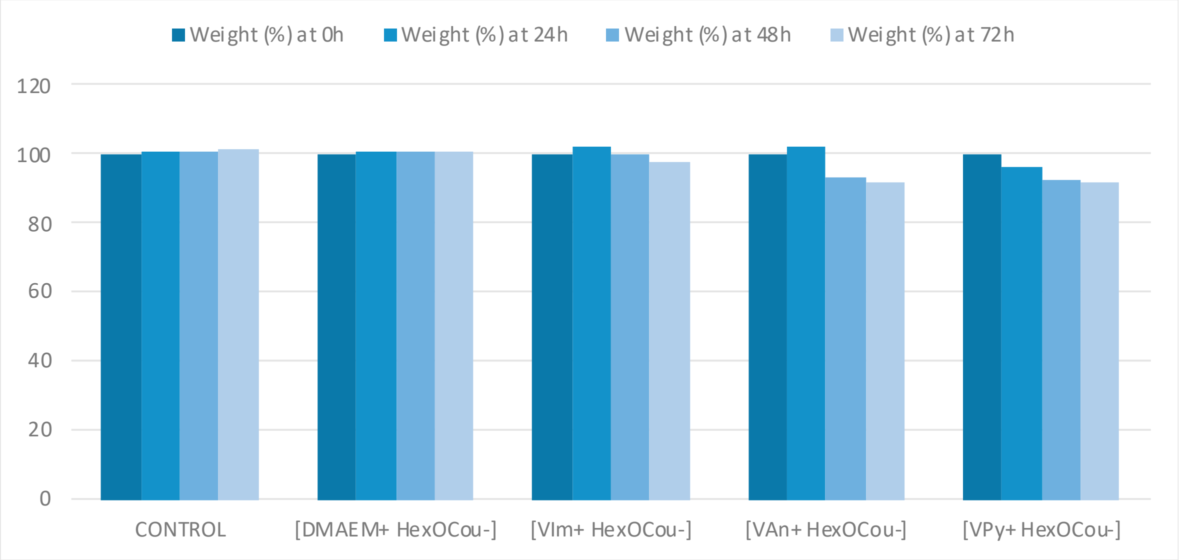

Figure S3. Swelling test of polymer coatings. Weight of polymers after 24h, 48h and 72h immersion in water.

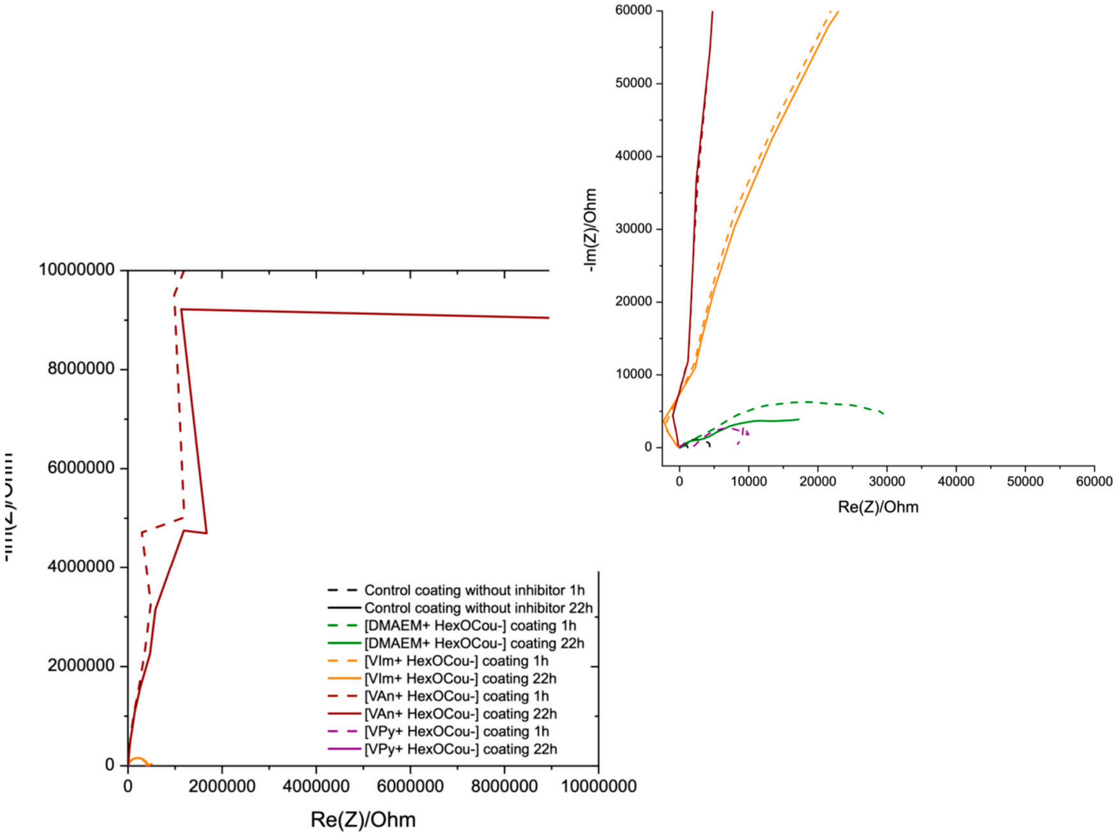

Figure S4. Nyquist plot of polymer coating on AS1020 mild steel containing 20% of [DMAEM+ HexOCou-] (green), 20% of [VIm+ HexOCou-] (orange), 20% of [VAn+ HexOCou-] (wine), 20% of [VPy+ HexOCou-] (purple) and control (black) immersed in 0.005M NaCl after 22h

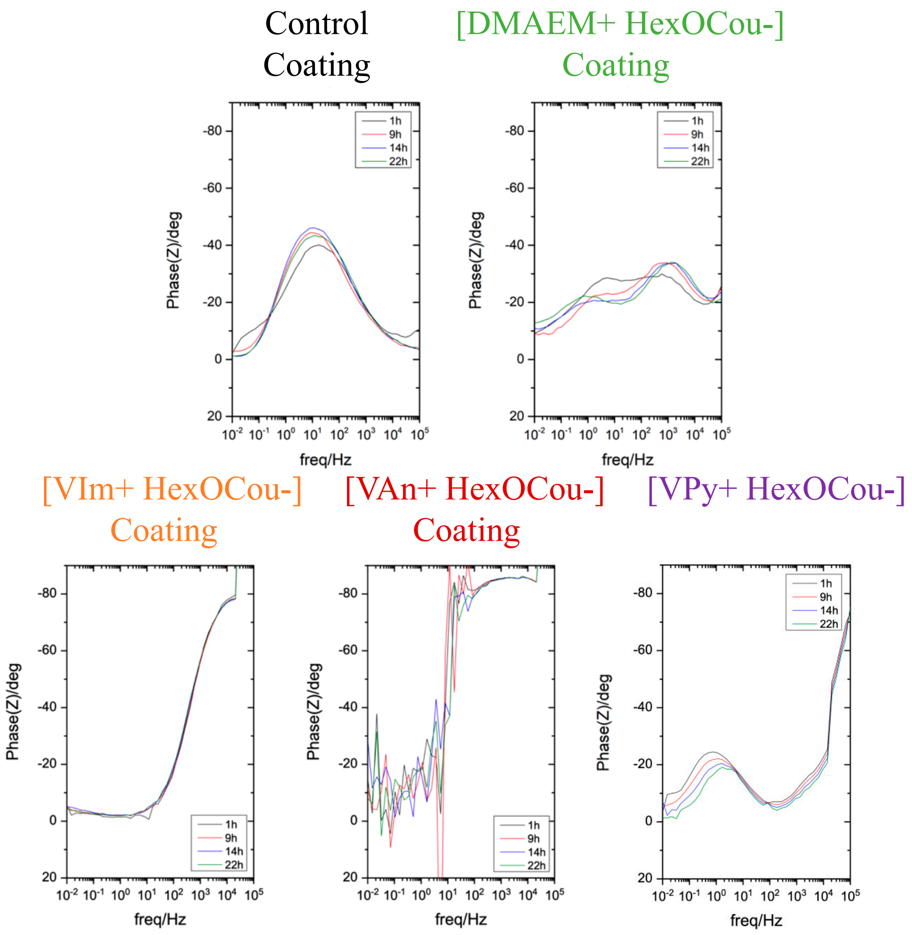

Figure S5. Electrochemical impedance spectra of different polymer coatings on AS1020 mild steel immersed in 0.005M NaCl at different immersion times: phase angle plots for control, inhibited coating containing 20% [DMAEM+ HexOCou-]; inhibited coating containing 20% [VIm+ HexOCou-]; inhibited coating containing 20% [VAn+ HexOCou-]; inhibited coating containing 20% [VPy+ HexOCou-]
